# Neurorehabilitation

**DOI:** 10.1007/s00115-024-01772-9

**Published:** 2024-11-26

**Authors:** Christian Dohle, Mareike Schrader

**Affiliations:** 1 P.A.N. Zentrum für Post-Akute Neurorehabilitation, Fürst Donnersmarck-Stiftung zu Berlin, Wildkanzelweg 28, 13465 Berlin, Deutschland; 2 Centrum für Schlaganfallforschung, Charité – Universitätsmedizin Berlin, Berlin, Deutschland; 3 Charité – Universitätsmedizin Berlin, Berlin, Deutschland

**Keywords:** International Classification of Functioning, Disability, and Health, Therapieprinzipen, Schlaganfall, Phasenmodell, Langzeitversorgung, International Classification of Functioning, Disability and Health, Treatment principles, Stroke, Phase model, Long-term care

## Abstract

Neurorehabilitation ist gekennzeichnet durch eine strukturierte, interdisziplinäre Zusammenarbeit verschiedener Professionen, orientiert an individuellen Teilhabezielen. Dabei müssen verschiedene Betrachtungsebenen von Funktionalität, Aktivität und Partizipation (International Classification of Functioning, Disability, and Health, ICF) berücksichtigt werden. Für die Rehabilitation von Störungen stehen bei verschiedenen Erkrankungen verschiedene evidenzbasierte Therapieverfahren zur Auswahl, die unterschiedlichen Wirkmechanismen zuzuordnen sind. Therapien müssen ausreichend intensiv sein. Das neurologische Phasenmodell beinhaltet neben der Akutbehandlung (Phase A) und der Phase D für weitestgehend selbstständige Patient:innen auch die Frührehabilitationsphase B und die Rehabilitationsphase C mit jeweils simultanen akut- und rehabilitationsspezifischen Behandlungsaufträgen. Zudem beinhaltet es im Langzeitverlauf die Phase E für die berufliche und soziale Teilhabe und die Phase F für die stationäre Langzeitversorgung schwerstbetroffener Patient:innen. Im ambulanten Sektor ist die Langzeitversorgung neurologisch Betroffener aufgrund mangelnder Möglichkeiten der Zusammenarbeit der beteiligten Disziplinen derzeit unzureichend und muss zwingend ausgebaut werden.

## Abstract (en)

Neurorehabilitation is characterized by a structured, interdisciplinary collaboration among various professional fields, focused on achieving individualized participation goals for patients. This process considers the different levels of the International Classification of Functioning, Disability, and Health (ICF), specifically function, activity and participation. Multiple evidence-based treatment procedures targeting specific mechanisms of action are available for the rehabilitation of disorders associated with various diseases. Treatment must be administered with sufficient intensity to be effective. The neurological phase model encompasses several stages of care ranging from acute treatment in phase A to phase D for patients who are largely independent. Early rehabilitation phase B and rehabilitation phase C combine acute and rehabilitation-specific tasks. Phase E supports long-term occupational and social participation, while phase F is dedicated to long-term care for severely affected patients. In the outpatient setting, the long-term care of neurologically affected patients remains insufficient due to a lack of interdisciplinary collaboration possibilities, highlighting an urgent need for expansion and improved integration between care providers.

## Lernziele

Nach Lektüre dieses Beitrags …

- haben Sie Kenntnis über das Konzept der Neurorehabilitation,
- haben Sie einen Überblick über relevante Leitlinien für die Neurorehabilitation,
- können Sie die wichtigsten Wirkmechanismen und Therapieprinzipien in der motorischen Rehabilitation nach einem Schlaganfall benennen,
- haben Sie Kenntnis über die Organisation der Neurorehabilitation in Deutschland (Phasenmodell),
- kennen Sie die Möglichkeiten der Umsetzung in der ambulanten Versorgung.

## Hintergrund

In den letzten Jahrzehnten hat die verbesserte neurologische Akutbehandlung zu einer Verringerung der Mortalität geführt [1]. Neue krankheitsmodifizierende medikamentöse Therapien haben die Überlebensdauer und damit aber auch die Gesamtbehinderungslast erhöht. In vielen Fällen bleiben alltagsrelevante Einschränkungen bzw. Behinderungen bestehen. Im Vergleich zu akutmedizinischen und medikamentösen Maßnahmen erfährt die symptomatische und vor allem therapeutische Behandlung jedoch deutlich geringere Aufmerksamkeit. Dabei weist dieser Sektor relevante Weiterentwicklungen auf. Wesentliche Grundzüge der Neurorehabilitation werden nachfolgend dargestellt.

### Fallbeispiel

Herr M. ist 58 Jahre alt, Berufskraftfahrer und erleidet zu Hause einen ischämischen Schlaganfall. Unmittelbar nach dem Ereignis ist sein linker Arm vollständig gelähmt und funktionslos, sein Bein mittelgradig gelähmt. Er ist auf den Rollstuhl angewiesen. Darüber hinaus hat er einen Neglekt nach links und eine Dysarthrie.

Von der Akutklinik wird Herr M. in eine Rehabilitationsklinik zur Phase-C-Rehabilitation überwiesen. Nach Aufnahmeuntersuchung und -gespräch werden gemeinsam Ziele definiert und ein Therapieplan erstellt. Begleitend erfolgt die Durchführung des noch ausstehenden Langzeit-Elektroenzephalogramms (EKG) und die Optimierung der antihypertensiven Medikation. Herr M. wohnt mit seiner Ehefrau im 3. Stock eines Mehrfamilienhauses ohne Fahrstuhl und möchte wieder in die Häuslichkeit zurückkehren. Daher liegt der Schwerpunkt der Physiotherapie vor allem auf dem Training des Gehens und des Treppensteigens. In der Ergotherapie erfolgt einerseits eine spezifische Armtherapie zur Verbesserung der Armfunktion, andererseits erlernt Hr. M. Kompensationstechniken, um den Alltag trotz Einschränkungen zu bewältigen. Die neuropsychologischen Therapien adressieren die kognitive Leistungsfähigkeit und das Neglekttraining. In der Logopädie erfolgen Übungen zur Verbesserung der Dysarthrie. Der Sozialdienst berät die Familie über mögliche Unterstützungsangebote im häuslichen Umfeld.

Nach drei Monaten wird Herr M. aus der Rehaklinik arbeitsunfähig entlassen, eine Erwerbsfähigkeit konnte für die nächsten 6 bis 12 Monaten nicht bescheinigt werden. Der Neglekt hat sich zurückgebildet und er ist in der Lage, die 3 Etagen im Wohnhaus selbständig zu bewältigen. Er benötigt für längere Strecken jedoch noch den Rollstuhl. Der Arm ist nach wie vor in seiner Funktion eingeschränkt und Herr M. ist bei vielen Aktivitäten des Alltags auf die Hilfe des externen Pflegedienstes bzw. seiner Frau angewiesen. Im ambulanten Umfeld bekommt Herr M. je 2‑mal pro Woche Ergo- und Physiotherapie.

## Was versteht man unter Neurorehabilitation?

Der Begriff „Rehabilitation“ wird national und international durchaus unterschiedlich interpretiert. Eine für Deutschland gut geeignete Definition der **Deutschen Vereinigung für Rehabilitation**

Deutschen Vereinigung für Rehabilitation

(DVfR; [2]) ist in Tab. 1 aufgeführt.

**Tab. 1:** Definition von Rehabilitation der Deutschen Vereinigung für Rehabilitation [2]

Rehabilitation fördert Menschen mit bestehender oder drohender Behinderung
Ziel ist die Stärkung körperlicher, geistiger, sozialer und beruflicher Fähigkeiten sowie die Selbstbestimmung und die gleichberechtigte Teilhabe in allen Lebensbereichen
Sie umfasst medizinische, therapeutische, pflegerische, soziale, berufliche, pädagogische oder technische Angebote einschließlich der Anpassung des Umfelds der Person
Rehabilitation ist ein an individuellen Teilhabezielen orientierter und geplanter, multiprofessioneller und interdisziplinärer Prozess
Sie achtet das Recht auf Selbstbestimmung

Das übergeordnete Ziel besteht demnach darin, dass Menschen mit Beeinträchtigungen in ihrer Lebensgestaltung so frei wie möglich werden. Der Kernpunkt der Rehabilitation beinhaltet das **koordinierte Zusammenwirken**

koordinierte Zusammenwirken

verschiedener Professionen und Fachdisziplinen unter **einheitlicher Zielsetzung**

einheitlicher Zielsetzung,

die gemeinsam mit den Betroffenen selbst und den an der Rehabilitation beteiligten Berufsgruppen erarbeitet wird.

### Merke

Neurorehabilitation ist gekennzeichnet durch eine strukturierte, interdisziplinäre Zusammenarbeit verschiedener Professionen, orientiert an individuellen Teilhabezielen.

Eine neurologische Rehabilitation kann bei **alltagsrelevanten Folgen**

alltagsrelevanten Folgen

aller neurologischen Erkrankungen indiziert und sinnvoll sein, v. a. bei Störungen der Motorik, der Sensorik, der Kognition, der Sprache und des Sprechens und des Schluckens. Für die Zielsetzung ist es aber wichtig, die Auswirkungen der Symptome auf die Lebensrealität der Betroffenen zu erfassen. Die Grundlage hierfür bildet das **ICF-Modell**

**Abb. 1:**
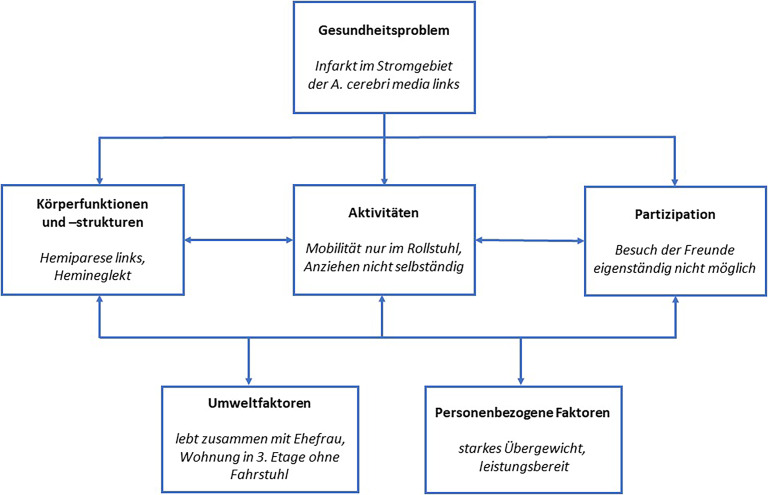
Modell der International Classification of Functioning, Disability and Health der World Health Organization (WHO) mit einer Konstellation aus dem Fallbeispiel

ICF-Modell

(International Classification of Functioning, Disability and Health, Abb. 1; [3]). Es unterscheidet danach, welche Folgen ein Gesundheitsproblem auf die Köperstrukturen, Körperfunktionen, Aktivitäten und Partizipation/Teilhabe hat. Darüber hinaus wird die Ausprägung der Folgen bedingt durch Wechselwirkungen mit Umweltfaktoren sowie personenbezogene Faktoren. Abb. 1 zeigt die Einordnung des Fallbeispiels in das ICF-Modell. Die ICF-Unterscheidung erscheint sehr formell, ist aber insbesondere in der **individuellen Zieldefinition**

individuellen Zieldefinition

und der Wahl der Therapieverfahren hilfreich (z. B. restaurativ oder kompensatorisch).

### Merke

In der Beurteilung der Neurorehabilitation müssen die verschiedenen Betrachtungsebenen der ICF berücksichtigt werden.

Das Aufgabenspektrum der Neurorehabilitation geht über die reine Anwendung therapeutischer Inhalte hinaus [4]. So spielt die **Überprüfung der Diagnostik**

Überprüfung der Diagnostik

in Zeiten immer kürzerer Liegezeiten im Krankenhaus eine prominente Rolle [5], ebenso wie die Behandlung der **Komorbiditäten**

Komorbiditäten.

Zudem treten während der wochen- bis monatelangen Liegezeiten immer wieder allgemeinärztliche Fragestellungen auf. Schließlich sichert die Neurorehabilitation einen belastbaren Übergang in die **poststationäre Nachsorge**

poststationäre Nachsorge,

was häufig nur mit erheblichem sozialmedizinischem Know-how und Engagement erreichbar ist.

## Wirkprinzipen therapeutischer Verfahren

Im Zentrum der Neurorehabilitation steht die **spezifische Therapie**

spezifische Therapie

der Krankheitssymptome. Dabei hat sich in den letzten Jahrzehnten sowohl das neurophysiologische Verständnis der Wirkprinzipien als auch die **Evidenzbasierung**

Evidenzbasierung

der angewandten Verfahren deutlich weiterentwickelt. Für viele therapeutische Inhalte existieren evidenzbasierte Leitlinien auf der Basis von Multicenterstudien bis hin zur Cochrane-Reviews. Dies ist besonders ausgeprägt für Symptome nach einem Schlaganfall, der typischerweise etwas mehr als die Hälfte der Behandlungsfälle in der Neurorehabilitation stellt. Eine Auswahl relevanter Leitlinien ist in Tab. 2 dargestellt.

**Tab. 2:** Relevante Leitlinien der Deutschen Gesellschaft für Neurologie/Deutschen Gesellschaft für Neurorehabilitation mit Aussagen zur Neurorehabilitation

Erkrankung	Symptome	Niveau	Leitlinie	Stand
Erkrankungsspezifisch				
Schlaganfall	Mehrere	S3	Schlaganfall [6]	29.02.2020
Schlaganfall	Armparese	S3	Rehabilitative Therapie bei Armparese nach Schlaganfall [7]	29.02.2020
Schlaganfall	Störung der Mobilität	S2e	Rehabilitation der Mobilität nach Schlaganfall (ReMoS) [8]	07.12.2015
Multiple Sklerose	Mehrere	S2k	Diagnose und Therapie der Multiplen Sklerose, Neuromyelitis-optica-Spektrum-Erkrankungen und MOG-IgG-assoziierten Erkrankungen – Living Guideline [9]	30.11.2023
Parkinson-Krankheit	Mehrere	S2k	Parkinson-Krankheit [10]	25.10.2023
Symptomspezifisch				
–	Koma und schwere Bewusstseinsstörung	S3	Neurologische Rehabilitation bei Koma und schwerer Bewusstseinsstörung im Erwachsenenalter [11]	23.12.2022
	Post-intensive-care-Syndrom	S2e	Multimodale Neurorehabilitationskonzepte für das Post-intensive-care-Syndrom (PICS) [12]	27.10.2022
	Störung der Sensomotorik	S2k	Rehabilitation von sensomotorischen Störungen [13]	01.02.2023
	Exekutive Dysfunktionen	S2e	Diagnostik und Therapie von exekutiven Dysfunktionen bei neurologischen Erkrankungen [14]	12.12.2019
	Gedächtnisstörungen	S2e 030-124	Diagnostik und Therapie von Gedächtnisstörungen bei neurologischen Erkrankungen [15]	26.02.2020
	Neglekt und Störungen der Raumkognition	S2k 030-126	Diagnostik und Therapie von Neglect und andere Störungen der Raumkognition [16]	01.03.2023

Wichtig ist, dass die spezifischen Therapieansätze den unterschiedlichen Krankheitsbildern mit unterschiedlichen Pathomechanismen folgen. So steht bei der Therapie zentraler Paresen, z. B. nach einem Schlaganfall, das **hochrepetitive Training**

hochrepetitive Training

an der jeweiligen Leistungsgrenze im Vordergrund. Das Training am individuellen Leistungsniveau erfolgt durch die Anpassung des Anforderungsniveaus, das sog. Shaping. Im Gegensatz dazu sind z. B. bei M. Parkinson als extrapyramidal-motorische Störung **volumenmodulierende Verfahren**

volumenmodulierende Verfahren

evidenzbasiert.

Die Kenntnisse verschiedener neurologischer Therapiemechanismen und -verfahren werden häufig im Rahmen einer **therapeutischen Berufsschulausbildung**

therapeutischen Berufsschulausbildung

nicht ausreichend differenziert erworben. Auch in den im niedergelassenen Bereich als Voraussetzung für die physiotherapeutische Verschreibungskategorie „KG-ZNS“ anerkannten **Weiterbildungen**

Weiterbildungen

Bobath, Vojta und propriozeptive neuromuskuläre Fazilitation (PNF) werden neue Erkenntnisse nicht ausreichend qualitätsgesichert vermittelt. Somit müssen bei einer ärztlichen Verschreibung therapeutischer Übungsverfahren deren Inhalte kritisch überprüft werden.

## Evidenzbasierte Therapieverfahren bei motorischen Störungen nach einem Schlaganfall

Die Grundlage für eine Verbesserung von Symptomen ist ein ausreichend intensives und **spezifisches Training**

spezifisches Training.

Inhaltlich steht gerade für die Therapie motorischer Störungen nach einem Schlaganfall ein besonders großer Evidenzkorpus von verschiedensten, teilweise sehr kleinteilig definierten Therapieverfahren zur Verfügung [7, 8]. Diese lassen sich nach den nachfolgend dargestellten **Wirkprinzipien**

**Abb. 2:**
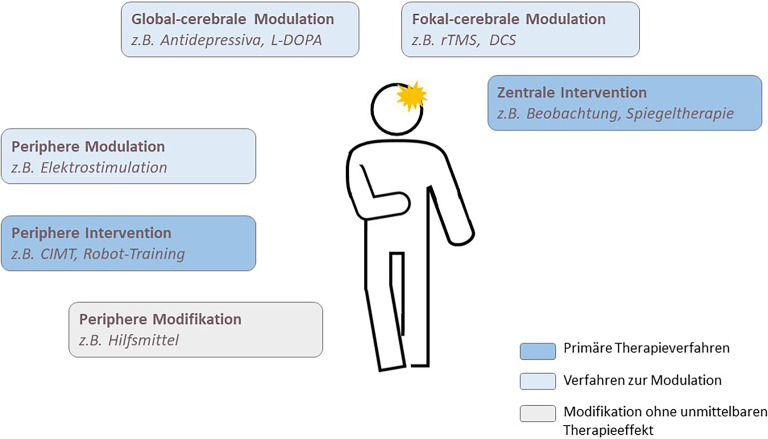
Klassifikation der Wirkprinzipien therapeutischer Verfahren. *CIMT* „constraint-induced movement therapy“, *DCS *Gleichstromstimulation, *L‑DOPA* Levodopa, *rTMS* repetitive transkranielle Magnetstimulation

Wirkprinzipien

einteilen und nach Bedarf kombinieren (Abb. 2). Dieses Schema lässt sich in ähnlicher Weise auch für andere Erkrankungen und Symptome übertragen.

### Periphere Intervention.

Besteht eine Lähmung einer Extremität, besteht der intuitive Therapieansatz in einem **aktiven Training**

aktiven Training

dieser Extremität, obwohl dieses vor allem über den Weg der propriozeptiven Afferenzen in Verbindung mit der zentralen Bewegungsrepräsentation wirkt. Dabei sollten die Bewegungen möglichst eigenständig initiiert werden, bei schwereren Paresen kann sie durch Therapeut:innen oder **Robotik**

Robotik

unterstützt werden. Bei mittelgradigen Armlähmungen kann die **„constraint-induced movement therapy“**

„constraint-induced movement therapy“

(CIMT) eingesetzt werden, bei der der Einsatz der betroffenen Seite durch Immobilisation der gesunden Seite forciert wird. Rein passive Bewegungen führen nicht zu einer Verbesserung der Bewegungsfähigkeit und sind höchstens zur Kontrakturprophylaxe sinnvoll.

### Zentrale Intervention.

Bei der zentralen Intervention wird eine direkte Aktivierung der Bewegungsrepräsentation angestrebt, z. B. durch Bewegungsbeobachtung, -spiegelung oder -vorstellung. Die Aktivierungsmuster hierbei sind denen bei aktiver Bewegungsausführung recht ähnlich [17] und wirken unabhängig von der Integrität afferenter Bahnen. Die **Spiegeltherapie**

Spiegeltherapie

wird insbesondere bei schweren Paresen empfohlen, bei der eigenständige Bewegungen nicht möglich sind, das Spiegelbild der nichtbetroffenen Extremität diese aber simuliert [18]. Bewegungsvorstellung oder Bewegungsbeobachtung haben noch keine ausreichende Evidenz für eine Leitlinienempfehlung.

Beide Verfahren können auch kombiniert werden, z. B. bei aktivem Training mit **visueller Rückkopplung**

visueller Rückkopplung

von Avataren unter Nutzung von Techniken der virtuellen Realität.

### Merke

Neuroplastizität zur Verbesserung von Defiziten kann entweder peripher (durch Beübung der betroffenen Extremität) oder direkt zentral (durch mentale Prozesse) gefördert werden.

Diese Verfahren können ergänzt werden mit Modulationstechniken, die keine eigenständige Wirksamkeit entfalten, aber die Wirkung der Intervention verstärken.

### Global-zerebrale Modulation.

Es gab mehrere Studien zum therapieunterstützenden Effekt von **Medikamenten**

Medikamenten

(z. B. Antidepressiva, Amphetamine oder L‑Dopa). Aktuell gibt es jedoch keine Substanz, die mit ausreichender Evidenz empfohlen werden kann. Auch Techniken zur Steigerung der Motivation wirken global verstärkend, sind aber derzeit noch nicht differenziert etabliert [19].

### Fokal-zerebrale Modulation.

Diese Verfahren zielen darauf ab, unmittelbar die Balance zwischen geschädigter und intakter Hemisphäre zu beeinflussen. Dabei kann sowohl die Erregbarkeit in der betroffenen, ipsiläsionalen Hemisphäre (periläsionell) gesteigert als auch die Aktivität der kontraläsionalen, intakten Hemisphäre reduziert werden [20]. Gängige Verfahren sind die repetitive transkranielle Magnetstimulation (**rTMS**

rTMS)

oder transkranielle Gleichstromstimulation (**tDCS**

tDCS),

für die je nach gewünschter Wirkung unterschiedliche Stimulationsparameter gewählt werden. In den Leitlinien wird aktuell lediglich die rTMS bei Armlähmungen empfohlen [7].

### Periphere Modulation.

Bei der peripheren Modulation bewirkt z. B. eine Elektrostimulation eine periphere Reizung, die nicht nur über propriozeptive oder taktile Afferenzen vermittelt wird. Bei geeigneter Programmierung überschwelliger Impulse kann die sog. **funktionelle Elektrostimulation**

funktionelle Elektrostimulation

(FES) konkrete Bewegungen wie das Greifen und Loslassen eines Gegenstandes auslösen und damit einen weiteren Therapieansatz ergänzen [21].

### Cave

Modulationsverfahren wie Medikamente oder nichtinvasive Hirnstimulation wirken nur in Kombination mit effektiven Interventionstechniken.

### Periphere Modifikation.

Schließlich können speziell bei motorischen Störungen Hilfsmittel wie z. B. **Gangorthesen**

Gangorthesen

eingesetzt werden, die die Funktionalität (z. B. bei einer spastischen Spitzfußstellung) unmittelbar verbessern. Ihr langfristiger neuroplastischer Effekt ist unklar.

Häufig wird als angewandte Methode das **Bobath-Konzept**

Bobath-Konzept

genannt. Dieses Konzept basiert allerdings auf verschiedenen neurophysiologischen und entwicklungsneurologischen Grundlagen und wird kontinuierlich weiterentwickelt [22]. Somit kann es keinem klaren Wirkprinzip zugeordnet werden. Es gilt auch nicht als evidenzbasiert.

### Merke

Für die Rehabilitation motorischer Störungen stehen viele verschiedene Therapieverfahren zur Auswahl, die unterschiedlichen Wirkmechanismen zuzuordnen sind. Therapien müssen ausreichend intensiv sein.

### Fallbeispiel Fortsetzung

Herr M. hat eine schwere armbetonte Hemiparese und kann nur mit Unterstützung ein paar Schritte gehen. Gemäß der aktuellen Leitlinie für den Arm [7] und die Mobilität [8] werden folgende Therapieverfahren angewandt: Für den Arm übt Herr M. intensiv mit der Spiegeltherapie**,** begleitend erfolgt ein roboterunterstütztes Armtraining. Im Verlauf zeigen sich bei Herrn M. erste Funktionen im Arm, sodass auf die „constraint-induced movement therapy“ (CIMT, „forced use“) gewechselt werden kann. Da vor allem die distalen Funktionsansätze durch eine deutliche Spastik überlagert sind, erhält Herr M. Botulinumtoxin injiziert. Für die Rehabilitation der Mobilität erhält Herr M. ein intensives Training mit dem Gangroboter. Im Verlauf kann er auf ein Laufband mit Gewichtsentlastung wechseln. Zur Unterstützung der Gangtherapie erhält Herr M. als Hilfsmittel eine Fußheberorthese und einen Vierpunkt-Gehstock. Er kann damit kurze Strecken laufen, ist aber für längere Strecken weiterhin auf den Rollstuhl angewiesen, dessen Handling in der Therapie eingeübt wird. Die einzelnen Therapieverfahren kommen mehrfach wöchentlich zur Anwendung. Die Entscheidung zum Wechsel der Therapieverfahren bei Verbesserung der Funktion werden in der wöchentlichen Teamkonferenz besprochen.

## Individualisierung des Rehabilitationsprozesses

Wie an den oben genannten Beispielen ersichtlich, spielt die Ausprägung der Defizite eine große Rolle in der Wahl der Therapieverfahren. Einschränkend ist allerdings zu sagen, dass das individuelle Ansprechen auf Therapien noch immer einer hohen Schwankungsbreite unterliegt, die noch nicht gut verstanden ist, zumal die zugrunde liegenden **Netzwerkinteraktionen**

Netzwerkinteraktionen

sowohl beim Gesunden als auch beim läsionierten Gehirn nicht hinreichend bekannt sind. Zudem muss in der Schwerpunktsetzung der individuelle Wunsch der Betroffenen berücksichtigt werden. Dies kollidiert aber mit dem Wunsch nach Erfassung des Rehabilitationserfolges mit unidimensionalen Skalen wie dem **Barthel-Index**

Barthel-Index

(BI). Mit dem BI werden vordefinierte Aktivitäten des täglichen Lebens bewertet, es kann ein Maximalscore von 100 Punkten erreicht werden. Eine **individuelle Priorisierung**

individuelle Priorisierung

der Ziele und deren Verlaufskontrolle kann beispielsweise mit dem international standardisierten Canadian Occupational Performance Measure (**COPM**

COPM)

durchgeführt werden. Eine Studie konnte zeigen, dass diese quantifizierte Beurteilung individueller Therapieziele Verbesserungen besser abbilden kann als globale Skalen [23].

### Cave

Globale Skalen können einen individualisierten Rehabilitationsprozess nur teilweise abbilden.

## Das neurologische Phasenmodell

Die Umsetzung der o. g. Inhalte in die Behandlungspraxis ist nicht trivial. In Deutschland sind Akutbehandlung und Rehabilitation auch leistungsrechtlich unterschiedlich organisiert. Da speziell bei neurologischen Patient:innen aufgrund ihrer multiplen Schädigungen diese klare Trennung häufig nicht zu ziehen ist, wurde das sog. neurologische Phasenmodell etabliert [24]. Es sieht vor, dass neben der reinen **Akutbehandlung**

Akutbehandlung

(Phase A) und der (auch teilstationär möglichen) **Rehabilitation**

Rehabilitation

schon in hohem Maße selbstständiger Patient:innen (Phase D) mit der Phase B und Phase C zwei **„Zwischenstufen“**

**Abb. 3:**
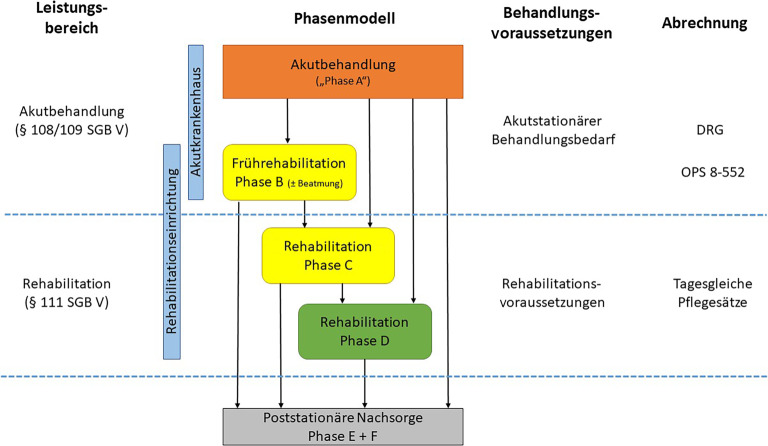
Schematische Darstellung des neurologischen Phasenmodells und seiner leistungsrechtlichen Einordnung. *DRG *„diagnosis related groups“, *OPS* Operationen- und Prozedurenschlüssel, *SGB* Sozialgesetzbuch

„Zwischenstufen“

bestehen, für die („hybrid“) sowohl akutmedizinische als auch rehabilitationsspezifische Behandlungsaufträge definiert sind (Abb. 3). In der Phase B sind Patient:innen noch schwer betroffen, teilweise intensivmedizinisch überwachungspflichtig, mit einer Trachealkanüle versorgt oder beatmet. Patient:innen der Phase C sind bewusstseinsklar und in der Lage, aktiv an den Therapien mitzuwirken, aber noch in hohem Maße auf pflegerische Unterstützung angewiesen.

### Merke

Das neurologische Phasenmodell beinhaltet neben der Akutbehandlung (Phase A) und der Phase D für weitegehend selbstständige Patient:innen auch die Frührehabilitationsphase B und die Rehabilitationsphase C mit jeweils simultanen akut- und rehabilitationsspezifischen Behandlungsaufträgen.

Zur Einpassung des Phasenmodells in die oben genannte Zweiteilung von Akutmedizin und Rehabilitation wurde die neurologisch-neurochirurgische **Frührehabilitation Phase B**

Frührehabilitation Phase B

(NNFR-B) als Krankenhausbehandlung definiert und die Phase C als Rehabilitationsbehandlung. Daher kann die NNFR‑B in zwei verschiedenen Settings stattfinden: entweder als Frührehabilitationsabteilung eines Akutkrankenhauses oder als Krankenhausabteilung (**„Fachklinik“**

„Fachklinik“)

im Verbund mit einer Rehabilitationsklinik der Phase C und D. In Deutschland sind beide Modelle weit verbreitet [25], quantitative Analysen zeigen in der Regel größere Patientenzahlen in Fachkliniken [26]. Da in der Krankenhausreform rein akutmedizinische Kriterien zur Anwendung kommen, ist die NNFR‑B aktuell gefährdet [27]. Zum Effekt der NNFR‑B liegen gute Daten aus Multicenterstudien [28] bis hin zu 5‑Jahres-Verläufen vor [29].

Die Einordnung der Phase B und C in unterschiedliche Leistungssegmente impliziert auch **unterschiedliche Zugangswege**

unterschiedliche Zugangswege.

Die NNFR‑B als Akutbehandlung kann entweder als **Direktverlegung**

Direktverlegung

oder nach vorheriger Kostenübernahme durch die Krankenkasse erfolgen. Als Krankenhausbehandlung ist sie gerechtfertigt bei akutstationärem Behandlungsbedarf, der nach den Mechanismen der Akutmedizin überprüft wird [30]. Für die Durchführung einer Phase C als Rehabilitationsbehandlung ist immer eine **vorherige Kostenübernahme**

vorherige Kostenübernahme

des jeweiligen Kostenträgers (in der Regel Krankenkasse) erforderlich.

Allgemeine Voraussetzungen für eine medizinische Rehabilitation sind Rehabilitationsbedürftigkeit, -fähigkeit und eine **positive Rehabilitationsprognose**

positive Rehabilitationsprognose.

Über diese allgemeinen Festlegungen hinaus sind im neurologischen Phasenmodell sehr präzise und **spezifische Ein- und Ausgangskriterien**

spezifische Ein- und Ausgangskriterien

für die Phase B und C definiert. Dabei sind die Kriterien für die Phasengrenzen nicht bundeseinheitlich, sondern länderspezifisch definiert [31]. In vielen Bundesländern wird dabei auf bestimmte Punktwerte des Barthel-Index als Hilfskonstrukt zurückgegriffen. Es ist offensichtlich, dass die gleichzeitige Anwendung verschiedener Kriterien immer wieder Inkongruenzen bei der Bewilligung bzw. Prüfung zur Folge hat [32].

### Merke

Für die Durchführung einer Phase C als Rehabilitationsbehandlung ist immer eine vorherige Kostenübernahme des jeweiligen Kostenträgers (in der Regel Krankenkasse) erforderlich.

## Ambulante Nachsorge

Da die Erholungskurve in der Neurorehabilitation häufig keinen klaren Endpunkt aufweist, sieht das neurologische Phasenmodell auch weitere Phasen im Langzeitverlauf vor. In der **Phase E**

Phase E

können nachgehende Leistungen speziell zur beruflichen oder sozialen Teilhabe verschiedener Leistungsträger untereinander kombiniert werden. Dieses Konzept ist jedoch derzeit in Deutschland nur in wenigen Modelleinrichtungen konsequent etabliert [23]. In der **Phase F**

Phase F

werden schwer- und schwerstbetroffene Patient:innen in speziellen Einrichtungen langzeitversorgt und stehen unter begleitender medizinischer und therapeutischer Versorgung.

### Merke

Das Phasenmodell beinhaltet im Langzeitverlauf die Phase E für die berufliche und soziale Teilhabe und die Phase F für die stationäre Langzeitversorgung schwerstbetroffener Patient:innen.

Somit ist das Konzept der Rehabilitation als koordiniertes Zusammenwirken verschiedener Berufsgruppen unter ärztlicher Leitung innerhalb der Rehabilitationskette der Phasen B bis D gut und auch in größerem Umfang qualitätsgesichert implementiert. Mit Verlassen dieser Phasen findet jedoch ein **Bruch der Behandlungskette**

Bruch der Behandlungskette

statt. Im **ambulanten Rahmen**

ambulanten Rahmen

der typischen haus- und fachärztlichen Versorgung fehlt die Möglichkeit des koordinierten Zusammenwirkens, da der Austausch der Leistungserbringer untereinander in der Regel nur bilateral (meist schriftlich) vorgesehen ist. Das Instrument der Teamsitzung mit Abstimmung der verschiedenen Professionen ist derzeit nur im Rahmen von Modellprojekten implementiert. Zudem weist der Heilmittelkatalog für ambulante Therapien verschiedenste Restriktionen auf, sowohl in der Therapieintensität als auch in der Therapiegestaltung (z. B. Verbot von Einzel- und Gruppentherapie am gleichen Tag, fehlende Vergütungsrelevanz der Anwendung evidenzbasierter Verfahren). Es gibt einzelne Praxen bzw. Einrichtungen, die es unter genauer Kenntnis der regulatorischen Rahmenbedingungen schaffen, für spezielle Symptomkomplexe zeitlich begrenzte, hochintensive Therapiephasen zu realisieren, die denen einer stationären Neurorehabilitation entsprechen. Insgesamt muss aber die Situation der längerfristigen neurorehabilitativen und therapeutischen Nachsorge in Deutschland als defizitär und unbefriedigend bezeichnet werden.

### Cave

Im ambulanten Sektor ist die Langzeitversorgung aufgrund mangelnder Möglichkeiten der Zusammenarbeit der beteiligten Disziplinen und der Finanzierung derzeit unzureichend und muss zwingend ausgebaut werden.

## Fazit für die Praxis

- Neurorehabilitation bezeichnet das koordinierte Zusammenwirken verschiedener Professionen unter einheitlicher Zielsetzung.
- Viele Verfahren der Neurorehabilitation für unterschiedliche Erkrankungen sind evidenzbasiert und in spezifischen Leitlinien verfügbar.
- In der Rehabilitation nach einem Schlaganfall als Modell für eine erworbene Hirnschädigung besteht das therapeutische Grundprinzip in hochfrequentem, symptomspezifischem Training an der jeweiligen Leistungsgrenze.
- In der deutschen Neurorehabilitation bestehen mit der Phase B und C zusätzliche Versorgungssegmente, in denen („hybrid“) sowohl akutmedizinische als auch rehabilitationsspezifische Behandlungsaufträge bestehen.
- Die Umsetzung des Rehabilitationsansatzes in der ambulanten Langzeitversorgung ist wünschenswert, aber kaum realisierbar.

## CME-Fragebogen

Welche Aussage trifft auf den Begriff der Rehabilitation zu?

Rehabilitation ist ein ausschließlich an motorischen Fähigkeiten orientierter und geplanter, multiprofessioneller und interdisziplinärer Prozess.

Rehabilitation fördert Menschen mit bestehender oder drohender Behinderung.

Rehabilitation schließt die Anpassung des Umfelds der Betroffenen nicht mit ein.

Für die Inhalte der Rehabilitationsmaßnahmen sind nur ärztliche Fragestellungen relevant.

Rehabilitation findet ausschließlich stationär statt.

Welche Voraussetzung gilt für eine medizinische Rehabilitation?

Pflegebedürftigkeit

Rehabilitationsbedürftigkeit

Volljährigkeit

Selbständigkeit in den Aktivitäten des täglichen Lebens

Negative Rehabilitationsprognose

Welche Aussage zu den Wirk- und Therapieprinzipien der Neurorehabilitation ist richtig?

Therapien sollten nicht intensiv sein, um eine Überlastung zu vermeiden.

Die Spiegeltherapie gehört zu der fokal-zerebralen Modulation.

Bei der peripheren Intervention wird eine direkte Aktivierung der mentalen Repräsentation von Bewegungen angestrebt.

Das Bobath-Konzept kann keinem klaren Wirkprinzip zugeordnet werden und gilt nicht als evidenzbasiert.

Patient:innen sollten immer nur mit einem Therapieverfahren behandelt werden, damit sich diese nicht gegenseitig beeinflussen.

Welches Therapieverfahren der Neurorehabilitation lässt sich der peripheren Intervention zuordnen?

Bewegungsbeobachtung

Spiegeltherapie

Rollstuhl

Transkranielle Gleichstromstimulation

Gangroboter

Welche Aussage zu Evidenzbasierung von Therapien in der Neurorehabilitation ist richtig?

Die meisten Therapien in der Neurorehabilitation beruhen rein auf intuitiven Konzepten.

Für viele Therapieverfahren liegen hochqualitative Wirksamkeitsstudien bis hin zu Cochrane-Reviews vor.

Therapieinhalte müssen individuell anhand von Originalliteratur erschlossen werden.

Bei der Auswahl der Therapieinhalte spielt die zugrunde liegende Diagnose in der Regel keine Rolle.

Therapien in der Neurorehabilitation können nicht standardisiert werden.

Herr Y. hat eine schwere Armlähmung. Welches Therapieverfahren ist insbesondere zur Funktionsanbahnung geeignet?

Constraint-induced movement therapy

Spiegeltherapie

Isolierte Hirnstimulation

Passive Durchbewegung

Injektion von Botulinumtoxin

Welche Aussage zum neurologischen Phasenmodell ist richtig?

Die Abgrenzung zwischen der Phase B (Frührehabilitation Krankenhausbehandlung) und der Phase C (Rehabilitation) ist eindeutig definiert.

Frührehabilitation (der Phase B) gilt als Rehabilitationsbehandlung.

In der Phase D sind Patient:innen weitgehend selbstständig und in den basalen Verrichtungen selbstversorgend.

Patient:innen in der Phase C sind bewusstseinsklar, können aber noch nicht aktiv an den Therapien mitwirken.

Die Rehabilitationszeit endet mit der Phase D.

Welche Besonderheit trifft auf die neurologisch-neurochirurgische Frührehabilitation Phase B (NNFR-B) zu?

Sie kann in Akutkliniken und in Rehabilitationsfachkliniken stattfinden.

Sie kann nicht als Direktverlegung erfolgen.

Sie setzt die Rehabilitationsfähigkeit der Patient:innen voraus.

Die Grenze zur Phase C ist bundeseinheitlich klar definiert.

Es bestehen nur akutmedizinische Behandlungsaufträge.

Frau X ist auf Beatmung über eine Trachealkanüle angewiesen, bewusstseinsklar und motorisch sehr eingeschränkt. Welcher Phase ist sie im neurologischen Phasenmodell zuzuordnen?

Phase A

Phase B

Phase C

Phase D

Phase F

Welche Aussage trifft auf die ambulante Versorgung der Neurorehabilitation zu?

Das neurologische Phasenmodell sieht auch weitere Phasen im Langzeitverlauf vor.

Leistungen speziell zur beruflichen oder sozialen Teilhabe verschiedener Leistungsträger stehen nicht im Fokus.

Das Konzept der Langzeitrehabilitation ist derzeit in Deutschland umfassend etabliert.

In der Phase F werden schwer- und schwerstbetroffene Patient:innen in speziellen Einrichtungen kurzfristig versorgt.

Der Austausch der Fachdisziplinen im der ambulanten Versorgung ist sehr gut ausgebaut und strukturiert.
